# Prevalence and factors associated with suicidal behavior among trans women in Rio de Janeiro, Brazil

**DOI:** 10.1371/journal.pone.0259074

**Published:** 2021-10-22

**Authors:** Ricardo de Mattos Russo Rafael, Emilia Moreira Jalil, Paula Mendes Luz, Cristiane Regina Vinissius de Castro, Erin C. Wilson, Laylla Monteiro, Michelle Ramos, Ronaldo Ismério Moreira, Valdiléa Gonçalves Veloso, Beatriz Gilda Jegerhorn Grinsztejn, Luciane de Souza Velasque

**Affiliations:** 1 Department of Public Health Nursing, School of Nursing, State University of Rio de Janeiro, Rio de Janeiro, Brazil; 2 Evandro Chagas National Institute of Infectious Diseases, Oswaldo Cruz Foundation, Rio de Janeiro, Brazil; 3 San Francisco Department of Public Health, Center for Public Health Research, San Francisco, California, United States of America; 4 Department of Epidemiology and Biostatistics, University of California, San Francisco, California, United States of America; 5 Department of Quantitative Methods, Federal University of Rio de Janeiro State, Rio de Janeiro, Brazil; Emory University, School of Public Health, UNITED STATES

## Abstract

**Background:**

Trans women face disproportionate burden of adverse health outcomes, including mental health issues. Very little is known about suicidal behavior among trans women in low- and middle-income settings, such as Brazil. We aimed to estimate the prevalence of lifetime suicidal behavior and to identify its associated factors among Brazilian trans women.

**Methods:**

This was a cross-sectional study conducted among 345 trans women living in Rio de Janeiro, Brazil. We examined the prevalence of suicidal behavior (ideation and suicide attempt) and its associated factors using stepwise backward Poisson regression analysis with robust variance.

**Results:**

Suicidal ideation was present among 47.25% of participants, and the prevalence of lifetime suicide attempt was 27.25%. Trans women with prior physical violence perpetrated by a family member had significantly higher prevalence of suicidal ideation (adjusted prevalence ratios [aPR]1.37), whereas those who reported sex work had lower prevalence ratio of suicidal ideation (aPR 0.76). Suicide attempt was significantly associated with living alone (aPR 1.48), physical violence by a casual partner (aPR 1.92), and sexual violence by a family member (aPR 1.69). Depression was significantly associated with both outcomes (aPR 1.90 for suicidal ideation and aPR 2.21 for suicide attempt).

**Conclusion:**

Suicidal behavior prevalence rates among Brazilian trans women were alarming and directly linked to violence and poor mental health. Effective mental health and public health policies addressing violence against trans women are urgently needed to prevent suicidal behavior among this highly vulnerable population.

## Introduction

Suicidal behavior (both ideation and attempt), defined as self-inflicted violence [1], is an important public health issue. Overall, 800,000 suicide deaths per year are estimated to occur worldwide, representing a rate of 10.5 deaths per 100,000 inhabitants. In Brazil, the suicide mortality rate increased from 4.8 deaths per 100,000 inhabitants in 2000 to 6.5 deaths per 100,000 in 2016 [2]. Suicide attempt has been associated with mental disorders, such as depressive syndromes [3–6]. Suicidal behavior has been found to increase in periods of stress, such as when people are facing financial problems, personal issues, or receive a severe disease diagnosis, among others. Moreover, social isolation and sexual violence contribute to causal pathways leading to suicidal behavior [7–10].

Vulnerable groups such as trans women have overlapping risks that may increase suicidal behavior [11–13]. In addition to the increased risk posed by mental health issues and crisis situations, trans women also face stigmas related to HIV and other sexually transmitted infections (STI) [14], low self-satisfaction with their bodies and/or gender expression [15], and high rates of minority stress related to their gender identity, such as discrimination, stigma, and violence [16, 17]. Suicide attempt rates among trans people ranged from 32% to 50% across countries, with no Brazilian estimates [18]. Although Brazil ranks first in violent deaths among trans people worldwide [19], there are limited data on psychological stress due to violence among Brazilian trans women. This group experience extremely high prevalences of discrimination and violence (psychological, physical and sexual) from community members, family, and intimate partners [14, 20, 21] in Brazil. Violence is a potential driver of mental health distress among trans women, which may lead to depression and other emotional and social problems. In addition, governmental violence may contribute to mental distress among trans women. The recent efforts to dismantle the rights of people who are LGBTQI+ in Brazil may be particularly destabilizing [22–24].

Brazilian data have shown that LGBTQI+ bodies, especially trans individuals, suffer systematic attempts to fit them into a binary and cisheteronormative gender culture [25]. Furthermore, social and racial inequalities, a major issue in Brazil, also impact the access to fundamental rights such as health, education and security. These disparities, combined to gender issues, affect the geographic territories occupied by trans people, such as the place that they live, buy, or gather together [26–28]. In addition, a substantial proportion of Brazilian trans women have been estimated to engage in sex work at some point in their lives [14, 29]. The current conservative tide in Brazil with unrelenting attacks to human rights [23] may further exacerbate the marginalization and vulnerability of trans women. Despite these aspects, the impact of interpersonal and government violence on mental health issues among trans women is understudied in Brazil. To fill this gap, we aimed to estimate the prevalence of suicidal behavior (suicidal ideation and suicide attempt) and to evaluate its associated factors among trans women in Rio de Janeiro, Brazil.

## Materials and methods

### Design and sample

This is a cross-sectional study on data gathered on *Transcender*, a Respondent-Driven Sampling survey conducted at the Oswaldo Cruz Foundation (FIOCRUZ) between August 2015 and January 2016, in Rio de Janeiro, Brazil. The Evandro Chagas National Institute of Infectious Diseases (INI) Institutional Review Board (IRB) reviewed and approved the study, and participants signed informed consent terms prior to any study procedure. Study procedures are described elsewhere [14]. Briefly, we enrolled participants aged 18 or older, who self-identified as trans women or a gender identity other than the male sex assigned at birth, and lived in the city of Rio de Janeiro or its metropolitan area. The current analysis enrolled all participants with valid results for the main outcomes. Participants answered face-to-face structured interviews administered by trained professionals, as well as performed HIV and STI testing, among others. All participants who had a mental health need identified, including but not limited to suicidal ideation or attempt, were referred to mental health assessment and care at INI-FIOCRUZ or to a referral health service of the Brazilian Public Health System.

### Measurements

The following questions (Y/N) assessed our main outcomes: "Have you ever thought about killing yourself?" (suicidal ideation) and “Have you ever tried to kill yourself?” (suicide attempt). Demographic covariates included age, self-declared race/color, schooling, self-reported gender identity, change in official documents, sexual orientation, marital status, housing situation, profession, and average household income. Housing situation encompassed three categories: own house, rented and unstable (shelter, somewhere as a favor, at work, or homeless). The average monthly household income was originally measured in R$ and converted to US$ (US$ 1.00 = R$ 3.89).

We evaluated data on social engagement in LGBTQI+ movements or organizations; discrimination, including at work, at home, and in health care services; binge drinking (6+ alcohol doses in one occasion); previous psychological, physical and sexual violence; body self-satisfaction; ever use of feminizing hormones, and current sex work. Current HIV status considered HIV rapid tests performed on the same day of the interview. We used the 10-item Center for Epidemiologic Studies Depression Scale (CES-D-10) to screen for depression. A 10+ score was deemed as positive for depression [30].

### Statistical analysis

We described the study population and estimated the prevalences and 95% confidence intervals (95%CI) of suicidal ideation and suicide attempt. We calculated crude Prevalence Ratios (cPR) using different Poisson regression models [31, 32] for each outcome. All variables with a p-value of 0.20 or less were included in the initial multivariable model. A stepwise backward Poisson regression analysis with robust variance was used to detect factors associated with the outcomes and to control for confounding factors. We reached the final model (all variables with a p-value <0.05) with the adjusted prevalence ratios (aPRs) by removing individually non-significant covariates. All analyses were performed using Stata SE version 15 [33].

## Results

All 345 participants enrolled in the *Transcender* study had valid data for our main outcomes and were included in the current analysis. The prevalences of suicidal ideation and suicide attempt were 47.25% (95%CI 42.00–52.55) and 27.25% (95%CI 22.78–32.21), respectively (Table 1). The sample characteristics show that 69.57% of the participants were aged 18 to 35 years, 75.07% self-reported as Black/Brown, 41.51% had up to 12 years of schooling, 94.49% were heterosexual, and 43.61% earned less than or equal to US$130 per month. 48.41% of participants were currently engaging in sex work. Only 25.22% of participants were very satisfied with their bodies. HIV prevalence was 41.23%. Rates of ever experiencing discrimination, psychological violence, physical and sexual violence were, respectively, 91.64%, 85.80%, 54.20%, and 47.54%. A positive depression screening occurred in 58.8% (Table 1).

**Table 1:** Prevalences of lifetime suicidal ideation and suicide attempt and sample characteristics of trans women in Rio de Janeiro, Brazil, 2015–2016 (n = 345).

Variables	N	%
**Lifetime suicidal ideation**	163	47.25
**Lifetime suicide attempt**	94	27.25
**Age**		
18–24	95	27.54
25–35	145	42.03
36–45	66	19.13
>45	39	11.30
**Self-declared race/color**		
White	79	22.90
Black/Brown	259	75.07
Others	7	2.03
**Schooling (years)**		
0–8	22	6.38
9–12	135	39.13
>12	188	54.49
**Changed name in legal documents**	9	2.61
**Sexual orientation**		
Heterosexual	326	94.49
Homosexual/Other	19	5.51
**Marital status**		
Single	278/344	80.81
Married or living with partner	66/344	19.19
**Living in RJ city**	262	75.94
**Housing situation**		
Own house	170	49.28
Rented house	99	28.70
Unstable	76	22.03
**Living alone**	113	32.75
**Current sex work**	167	48.41
**Average household income (US$)**		
≤130	140/321	43.61
131–260	109/321	33.96
>260	72/321	22.43
**Social engagement**	94	27.25
**Ever discrimination**	307/335	91.64
**Binge drinking** ^**c**^	303	87.83
**Psychological violence**	296	85.80
**Physical violence**	187	54.20
By main partner	55	15.94
By casual partner	20	5.80
By family member/relative	59	17.10
By co-worker	25	7.25
By friends or acquaintances	44	12.75
By client	60	17.39
By police	55	15.94
By unknown person	119	34.49
**Sexual violence**	164	47.54
By main partner	11	3.19
By casual partner	8	2.32
By family member/relative	44	12.75
By co-worker	2	0.58
By friends or acquaintances	47	13.62
By client	31	8.99
By police	21	6.09
By unknown person	56	16.23
**Body self-satisfaction**		
Very satisfied	87	25.22
A little bit satisfied	147	42.61
A little unsatisfied	86	24.93
Very unsatisfied	25	7.25
**Ever hormone use**	325	94.20
**Depression** ^**d**^	203	58.84
**HIV-positive status**	141/345	41.23

In the initial adjustment model, sexual orientation was positively associated with lifetime suicidal ideation, and current sex work was negatively associated with the outcome (Table 2). Living alone and sexual violence by family were factors positively associated with lifetime attempted suicide (Table 3). Positive depression screening was positively associated with both outcomes.

**Table 2:** Crude and initial model of adjusted prevalence ratio of lifetime suicidal ideation among trans women in Rio de Janeiro, Brazil, 2015–2016.

Variables	PR^a^ (95%CI)	p-value	aPR^b^ (95%CI)	p-value
**Age**				
18–24	Reference	-	-	-
25–35	1.13 (0.87–1.47)	0.339	-	-
36–45	0.83 (0.58–1.20)	0.325	-	-
>45	0.75 (0.47–1.21)	0.248	-	-
**Self-declared race/color**				
White	Reference	-	-	-
Black/Brown	0.87 (0.67–1.11)	0.279	-	-
Others	1.38 (0.82–2.30)	0.224	-	-
**Schooling (years)**				
0–8	0.78 (0.51–1.21)	0.275	-	-
9–12	0.90 (0.60–1.36)	0.639	-	-
>12	Reference	-		
**Changed name in legal documents**	0.70 (0.43–1.12)	0.144	0.68 (0.37–1.27)	0.230
**Sexual orientation**				
Heterosexual	Reference	-	Reference	-
Homosexual/Other	1.48 (1.07–2.06)	**0.018**	1.42 (1.01–2.01)	**0.046**
**Marital status**				
Single	Reference	-	-	-
Married or living with partner	1.16 (0.89–1.50)	0.263	-	-
**Living in RJ city**	0.93 (0.70–1.21)	0.584	-	-
**Housing situation**				
Own house	Reference	-	Reference	-
Rented house	1.31 (1.01–1.70)	**0.036**	1.18 (0.91–1.54)	0.212
Unstable	1.32 (1.00–1.75)	**0.044**	1.10 (0.83–2.01)	0.505
**Living alone**	1.07 (0.85–1.35)	0.545		
**Current sex work**	0.80 (0.63–1.00)	0.058	0.78 (0.62–0.99)	**0.049**
**Average household income (US$)**				
≤130	1.11 (0.82–1.51)	0.481	-	-
131–260	1.01 (0.72–1.40)	0.946	-	-
>260	Reference	-	-	-
**Social engagement**	0.76 (0.60–0.95)	**0.015**	0.89 (0.70–1.13)	0.356
**Ever discrimination**	2.34 (0.97–5.64)	0.057	1.67 (0.88–3.17)	0.120
**Binge** ^**c**^	1.05 (0.73–1.50)	0.785	-	-
**Psychological violence**	1.42 (0.95–2.12)	0.086	0.99 (0.66–1.48)	0.972
**Physical violence**	1.38 (1.08–1.74)	**0.008**	1.01 (0.73–1.41)	0.927
By main partner	1.49 (1.18–1.88)	**0.001**	1.16 (0.99–1.54)	0.289
By casual partner	1.52 (1.11–2.08)	**0.008**	1.09 (0.74–1.63)	0.645
By family member/relative	1.52 (1.21–1.91)	**<0.001**	1.22 (0.91–1.64)	0.177
By co-worker	1.02 (0.66–1.55)	0.937	-	-
By friends or acquaintances	1.18 (0.87–1.59)	0.271	-	-
By client	1.25 (0.97–1.61)	0.085	0.90 (0.66–1.22)	0.492
By police	1.09 (0.82–1.46)	0.542	-	-
By unknown person	1.26 (1.00–1.57)	**0.041**	1.02 (0.77–1.36)	0.876
**Sexual violence**	1.26 (1.01–1.58)	**0.041**	0.99 (0.77–1.25)	0.914
By main partner	1.16 (0.67–2.01)	0.597	-	-
By casual partner	0.79 (0.32–1.95)	0.609	-	-
By family member/relative	1.18 (0.88–1.59)	0.271	-	-
By co-worker	2.13 (1.90–2.38)	**<0.001**	1.41 (0.84–2.36)	0.191
By friends or acquaintances	1.14 (0.85–1.54)	0.358	-	-
By client	1.33 (0.98–1.81)	0.062	1.26 (0.92–1.73)	0.153
By police	0.69 (0.37–1.28)	0.242	-	-
By unknown person	1.11 (0.84–1.48)	0.442	-	-
**Body self-satisfaction**				
Very satisfied	Reference	-	Reference	-
A little bit satisfied	1.23 (0.90–1.70)	0.191	1.09 (0.79–1.49)	0.590
A little unsatisfied	1.35 (0.96–1.89)	0.084	1.04 (0.73–1.48)	0.793
Very unsatisfied	1.79 (1.22–2.62)	**0.003**	1.34 (0.92–1.95)	0.125
**Ever hormone use**	0.94 (0.59–1.48)	0.795	-	-
**Depression** ^**d**^	1.95 (1,14–2.56)	**< .001**	1.62 (1.22–2.15)	**0.001**
**HIV-positive status**	0.90 (0.71–1.14)	0.388	-	-

**(a)** PR: prevalence ratio

**(b)** aPR: adjusted PR (initial model), 95%CI: 95% confidence interval

**(c)** 6+ alcohol doses in one occasion

**(d)** CES-D 10 [30].

**Table 3:** Crude and initial model of adjusted prevalence ratio of lifetime attempted suicide among trans women in Rio de Janeiro, Brazil, 2015–2016.

Variables	PR^a^ (95%CI)	p-value	aPR^b^ (95%CI)	p-value
**Age (years)**				
18–24	Reference	-	Reference	-
25–35	1.25 (0.83–1.89)	0.271	1.20 (0.81–1.77)	0.359
36–45	0.86 (0.49–1.50)	0.607	0.80 (0.45–1.42)	0.452
>45	0.58 (0.26–1.31)	0.194	0.56 (0.24–1.31)	0.184
**Self-declared race/color**				
White	Reference	-	-	-
Black/Brown	1.01 (0.67–1.54)	0.938	-	-
Others	1.61 (0.63–4.09)	0.315	-	-
**Schooling (years)**				
0–8	0.95 (0.49–1.85)	0.891	-	-
9–12	0.77 (0.39–1.48)	0.437	-	-
>12	Reference	-	-	-
**Changed name in legal documents**	2.49 (0.39–15.98)	0.336	-	-
**Sexual orientation**				
Heterosexual	Reference	-	-	-
Homosexual/Other	1.17 (0.59–2.32)	0.654	-	-
**Marital status**				
Single	Reference	-	-	-
Married or living with partner	1.21 (0.80–1.81)	0.353	-	-
**Living in RJ city**	0.85 (0.55–1.31)	0.467		
**Housing situation**				
Own house	Reference	-	Reference	-
Rented house	1.53 (1.01–2.29)	**0.042**	0.98 (0.64–1.50)	0.933
Unstable	1.61 (1.05–2.47)	**0.027**	1.10 (0.71–1.72)	0.655
**Living alone**	1.52 (1.08–2.14)	**0.016**	1.60 (1.12–2.27)	**0.008**
**Current sex work**	0.90 (0.63–1.27)	0.546	-	-
**Average household income (US$)**				
≤130	1.15 (0.72–1.83)	0.538	-	-
131–260	0.94 (0.56–1.55)	0.807	-	-
>260	Reference	-	-	-
**Social engagement**	0.69 (0.49–0.98)	**0.040**	0.83 (0.57–1.21)	0.347
**Ever discrimination**	5.46 (0.80–37.19)	**0.083**	2.35 (0.60–9.18)	0.219
**Binge drinking** ^**c**^	1.49 (0.77–2.85)	0.229	-	-
**Psychological violence**	1.56 (0.84–2.90)	**0.156**	0.76 (0.39–1.45)	0.409
**Physical violence**	1.56 (1.08–2.25)	**0.017**	1.23 (0.72–2.11)	0.447
By main partner	1.61 (1.10–2.35)	**0.014**	0.93 (0.59–1,47)	0.761
By casual partner	2.15 (1.39–3.34)	**0.001**	1.59 (0.83–3.06)	0.158
By family member/relative	1.57 (1.07–2.29)	**0.019**	0.87 (0.55–1.38)	0.551
By co-worker	1.87 (1.19–2.93)	**0.006**	1.25 (0.65–2.41)	0.497
By friends or acquaintances	1.40 (0.90–2.17)	**0.127**	0.96 (0.60–1.51)	0.854
By client	1.28 (0.85–1.93)	0.231	-	-
By police	1.42 (0.95–2.12)	**0.084**	0.94 (0.60–1.46)	0.779
By unknown person	1.29 (0.91–1.82)	**0.153**	0.85 (0.55–1.32)	0.476
**Sexual violence**	1.70 (1.19–2.42)	**0.004**	1.29 (0.84–2.71)	0.233
By main partner	1.70 (0.87–3.34)	**0.120**	1.51 (0.84–2.72)	0.164
By casual partner	1.39 (0.56–3.46)	0.481	-	-
By family member/relative	1.85 (1.26–2.70)	**0.002**	1.56 (1.01–2.43)	**0.046**
By co-worker	1.84 (0.45–7.46)	0.391	-	-
By friends or acquaintances	1.30 (0.83–2.02)	0.244	-	-
By client	1.20 (0.70–2.07	0.499	-	-
By police	1.05 (0.52–2.12)	0.887	-	-
By unknown person	1.22 (0.79–1.87)	0.357	-	-
**Body self-satisfaction**				
Very satisfied	Reference	-	Reference	-
A little bit satisfied	1.41 (0.87–2.29)	**0.160**	1.26 (0.78–2.03)	0.336
A little unsatisfied	1.34 (0.79–2.30)	0.272	1.02 (0.57–1.82)	0.940
Very unsatisfied	1.74 (0.89–3.38)	**0.103**	1.11 (0.58–2.11)	0.738
**Ever hormone use**	1.38 (0.56–3.38)	0.476	-	-
**Depression** ^**d**^	2.43 (1.57–3.76)	**<0.001**	1.90 (1.18–3.06)	**0.008**
**HIV-positive status**	0.96 (0.67–1.37)	0.818	-	-

**(a)** PR: prevalence ratio

**(b)** aPR: adjusted PR (initial model), 95%CI: 95% confidence interval

**(c)** 6+ alcohol doses in one occasion

**(d)** CES-D 10 [30].

In the final adjusted model, trans women currently engaging in sex work had lower PR of suicidal ideation compared to those not on current sex work (p-value <0.015). Ever suffering physical violence perpetrated by a family member or relative was positively associated with suicidal ideation (p-value 0.006). Transwomen who were currently living alone (p-value 0.019), those reporting ever physical violence from a casual partner (p-value 0.002) and ever sexual violence from a family member or relative (p-value 0.005) had significantly higher PR of suicide attempt in the final adjusted model. Positive depression screening was positively associated with suicidal ideation and suicide attempt (Table 4).

**Table 4:** Final model of predictors of lifetime suicidal ideation and attempted suicide among trans women in Rio de Janeiro, Brazil, 2015–2016.

Variables	aPR^a^ (95%CI)	p-value	aPR^a^ (95%CI)	p-value
**Living alone**	-	-	1.48 (1.06–2.06)	**0.019**
**Current sex work**	0.76 (.61 - .95)	**0.015**	-	-
**Physical violence**	-	-	-	-
By casual partner	-	-	1.92 (1.28–2.88)	**0.002**
By family member/relative	1.37 (1.09–1.71)	**0.006**	-	-
**Sexual violence**	-	-	-	-
By family member/relative	-	-	1.69 (1.17–2.44)	**0.005**
**Depression** ^**b**^	1.90 (1.44–2.51)	**<0.001**	2.21 (1.42–3.43)	**<0.001**

**(a)** aPR: adjusted PR, 95%CI: 95% confidence interval

**(b)** CES-D 10 [30].

## Discussion

Experiences of suicidal ideation and suicide attempt were alarmingly high among trans women living in Rio de Janeiro, Brazil. Almost half of the study population reported suicidal ideation, and 27.2% of trans women had ever attempted suicide. Recently, Malta et al. observed that suicidality was frequent in focus group discussion conducted with 50 individuals from sexual and gender minorities in Rio de Janeiro, Brazil [22]. To our knowledge, ours are the first quantitative results on suicidal behavior among Brazilian trans women and contribute to fill the dearth of data for this highly vulnerable, marginalized population in Brazil.

Our findings are consistent with data among trans people from other settings. A systematic review described prevalences of suicidal ideation ranging from 37–81% and suicide attempt from 18–41% among trans women; most studies identified that at least 50% of participants reported suicidal ideation and 30% reported a suicide attempt [34]. Another recent systematic review confirmed the high prevalences of suicidal behavior among trans women [35]. A Latin-American study observed that 20% and 30% of trans women in Argentina and in the Dominican Republic ever attempted suicide [36, 37].

Our data show that suicidal behavior among trans women is much higher than in the general population. In a survey conducted in 17 countries, the prevalences of suicidal ideation and suicide attempt in the overall population were, respectively, 9.2% and 2.7% [38]. LGBTQI+ people have higher suicide risk compared to the overall population, and trans people have two-times the risk of suicide behavior compared to other LGBTQI+ groups [39–41]. In addition, the World Health Organization (WHO) global suicide estimates [5, 42] are much lower than those observed among trans women. The disproportionately high prevalences of suicide attempt and suicidal ideation among Brazilian trans women reach epidemic proportions requiring an urgent targeted public health response.

We identified an association between depression and suicidal behavior, which is consistent with the literature. Depression is a main predictor of suicidal behaviors in different populations [34–36, 43–48], including trans women. Depression is also a major mental health problem among trans women. More than half of participants in a trans-specific cohort in Rio de Janeiro, Brazil, had a positive depression screening at enrollment [49]. This rate is higher than observed among U.S. trans women [50–52] and among cisgender women, who had depression prevalences ranging between 13.2 and 20.2% [53, 54]. Worldwide, 4.4% of people are estimated to have depression [2]. The high rates of mental health issues, including depression, among trans women are closely connected to the adversity they face in their daily lives. The pervasive discrimination against trans women in traditional contexts that deny gender identity diversity contributes to internalized transphobia and poor social determinants of health preventing access to education, employment, housing and fundamental human rights [23].

We also found an association between suicidal behavior and physical and sexual violence from partners and family members, which is consistent with other studies [55, 56]. Violence experience may trigger mental health issues, including depression. In addition, the synergistic interaction of physical and sexual violence with daily discrimination likely exceeds trans women’s coping capacity and heightens risk for depression and anxiety [56–58].

In our study, trans women living alone had significantly higher prevalence of suicide attempt. Social isolation has been associated with suicidal behavior [59, 60]. Long-lasting loneliness is associated with adverse health outcomes, including depression and suicidal behavior [61]. Levi et al. [62] analyzed the relationship between loneliness and suicidal behavior and identified that the levels of intimate communication were significantly lower among lonely individuals when compared to controls. These authors also found that people with high suicide attempt potential had lower levels of interaction and were lonelier. Limited social interaction may also affect the regulation of pain and psychological distress and may pose a higher risk for suicidal behavior [63].

Denial of basic human rights is common among Brazil trans women [23]. This may ultimately contribute to the high suicidality found in this analysis. Trans women in Brazil experience high exposure to violence and low access to services that affirm their gender, such as access to changes on official documents [14], which increase suicidal behaviors [37, 64]. These barriers represent systematic denials of social rights and are potential consequences of a binarist and cisheteronormative culture [65].

The negative association between current sex work and lifetime suicidal ideation identified in our analysis contrasts with results from other studies. U.S. data showed higher prevalence of suicide attempt in trans women who were sex workers [66]. In Brazil, trans women often rely on sex work as their sole income option. In our sample, 80% of the participants had ever engaged in sex work, although only ~48% currently reported sex work. Despite being a vulnerable group worldwide, Brazilian trans women live in disproportionate deleterious conditions that exacerbate their vulnerabilities. Brazil ranks first countries in trans women murders worldwide [19].

A stigmatizing, gender binary and cisheteronormative culture, combined with public policies that notably overlook trans people [23], decrease job opportunities and exclude trans women from the formal labor market. Sex work may be an indirect measure of income among trans women, and those not engaging in sex work may actually face a worst economic situation, which could partially explain its association with suicidal behavior. In addition, sex work may potentially be perceived as part of gender affirmation and contribute to reduced psychological suffering despite the oppression, discrimination and risks related to it. As it directly involves the recognition of trans bodies and the desire for them, sex work may acting as an element of social support, as hypothesized by Sevelius [67]. Prior studies have indicated passing (hereby considering the degree to which trans people are socially perceived as the gender with which they identify) as an important element for decreased violence and social inclusion [68]. Nevertheless, understanding these complex interactions needs a more in-depth examination in future research among trans women.

This study has some limitations. First, its cross-sectional design inherent has possible reverse causality bias. In addition, we did not use specific scales to measure violence and suicidal behavior. Also, the *Transcender* study was not specifically designed to assess suicide [14] and did not address important aspects related to suicide, such as access to mental health services. Finally, our sample may not represent all trans women, as such our results may not be generalizable to the whole population. Nevertheless, we identified alarming rates of suicidal behavior among Brazilian trans women, directly linked to violence and poor mental health.

Although the Brazilian health system is universal and every person has the right to access its health units, trans people have countless barriers to accessing health in the country [69]. Discrimination constantly threatens trans women’s rights, including access to health. Our findings shed some light on the intricated relations between socioeconomic aspects, as well as the multiple expressions of structural violence [70], and mental health issues among trans women. Strategies to reduce suicide in trans people should focus not only on qualified care and effective access to the health system, but also on providing social support and ensuring the rights of trans women. Effective mental health policies and interventions to address violence against trans women are urgently needed to prevent suicidal behavior with the effective inclusion of this population group in the health system.

## Supporting information

S1 File (XLSX)
